# Iterative Decomposition of Water and Fat with Echo Asymmetry and Least-Squares Estimation (IDEAL) Magnetic Resonance Imaging as a Biomarker for Symptomatic Multiple Myeloma

**DOI:** 10.1371/journal.pone.0116842

**Published:** 2015-02-23

**Authors:** Miyuki Takasu, Yoko Kaichi, Chihiro Tani, Shuji Date, Yuji Akiyama, Yoshiaki Kuroda, Akira Sakai, Kazuo Awai

**Affiliations:** 1 From the Department of Diagnostic Radiology, Graduate School of Biomedical Sciences, Hiroshima University, Hiroshima, Japan; 2 Department of Hematology and Oncology, Research Institute for Radiation Biology and Medicine, Hiroshima University, Hiroshima, Japan; 3 Department of Radiation Life Sciences, Fukushima Medical University School of Medicine, Fukushima, Japan; Wayne State University, UNITED STATES

## Abstract

**Introduction:**

To evaluate the effectiveness of iterative decomposition of water and fat with echo asymmetry and least-squares estimation (IDEAL) magnetic resonance imaging (MRI) to discriminate between symptomatic and asymptomatic myeloma in lumbar bone marrow without visible focal lesions.

**Materials and Methods:**

The lumbar spine was examined with 3-T MRI in 11 patients with asymptomatic myeloma and 24 patients with symptomatic myeloma. The fat-signal fraction was calculated from the ratio of the signal intensity in the fat image divided by the signal intensity of the corresponding ROI in the in-phase IDEAL image. The *t* test was used to compare the asymptomatic and symptomatic groups. ROC curves were constructed to determine the ability of variables to discriminate between symptomatic and asymptomatic myeloma.

**Results:**

Univariate analysis showed that β2-microglobulin and bone marrow plasma cell percent (BMPC%) were significantly higher and fat-signal fraction was significantly lower with symptomatic myeloma than with asymptomatic myeloma. Areas under the curve were 0.847 for β_2_;-microglobulin, 0.834 for fat-signal fraction, and 0.759 for BMPC%.

**Conclusion:**

The fat-signal fraction as a biomarker for multiple myeloma enables discrimination of symptomatic myeloma from asymptomatic myeloma. The fat-signal fraction offers superior sensitivity and specificity to BMPC% of biopsy specimens.

## Introduction

Asymptomatic multiple myeloma is an asymptomatic proliferative disorder of plasma cells with high risk of progression to symptomatic multiple myeloma. This risk is estimated at 10% per year during the first 5 years [1–3]. Progression represents the most serious clinical problem of patients with asymptomatic myeloma. Several groups [2, 4, 5] have therefore proposed different score systems to distinguish between patients with slowly and rapidly progressive asymptomatic myeloma. However, these systems require complex or expensive tools such as multiparametric flow cytometry of bone marrow plasma cells or equipment for comparative genomic hybridization, limiting the feasibility of such approaches in clinical practice.

Considering the high rate of progression of asymptomatic myeloma to symptomatic myeloma, several reports have analyzed the value of early treatment. However, none of these studies have demonstrated any clear benefit for patients in terms of overall survival when treated in the absence of symptoms [6–9]. At present, in the absence of reliable elements to predict disease progression, the standard of care for asymptomatic myeloma patients remains close follow-up without treatment until myeloma symptoms develop [3].

Regarding the relationship between findings on magnetic resonance imaging (MRI) and the prognosis of multiple myeloma, Stäbler et al. [10] assessed the correlation between MR infiltration pattern, biopsy findings, and a clinical staging system. They identified five infiltration patterns using T1-weighted and opposed gradient-recalled echo images and demonstrated that the infiltration pattern on unenhanced MRI correlated with the Durries/Salmon staging system. They concluded that high-grade plasma cell infiltration with replacement of fat cells caused a pronounced decrease in signal intensity on T1-weighted imaging. Furthermore, they used the opposed-phase gradient-recalled echo sequence on the basis that a shift in the fat-water ratio results in increased signal intensity, allowing bone-marrow infiltrating processes to be detected using opposed-phase sequences [10]. However, this kind of qualitative approach lacks objectivity. For example, they differentiated MR infiltration patterns by signal intensity on T1-weighted images and opposed-phase gradient-recalled echo images in correlation to the intervertebral discs, which might have been subjects to degenerative change. Therefore, this could have had an influence on the result which showed that MR infiltration pattern had correlation to the clinical staging system.

Separation of fat and water on MR images based on their chemical shift was originally discussed by Dixon in 1984 [11]. The original two-point technique with one in-phase and one opposed-phase image was later extended to multi-point methods [12–14]. Many spin-echo (SE) or fast SE (FSE) approaches acquire three echoes shifted symmetrically about the SE, creating time-dependent phase shifts caused by water-fat chemical shift [13,15,16]. This work demonstrates that symmetrically acquired echoes cause artifacts that degrade image quality. The noise performance of any water-fat separation method is dependent on the proportion of water and fat within a voxel, and the position of echoes relative to the SE. IDEAL combines asymmetrically acquired echoes with an iterative least-squares decomposition algorithm to maximize noise performance [17]. In recent years, substantial efforts have been made to use the IDEAL technique not only to separate water and fat, but also to obtain a quantitative measure of fat content [17–21]. Clinical use of this technique has mainly focused on the liver [19–21]. Recently, however, spinal infiltration by multiple myeloma has been investigated noninvasively using the IDEAL sequence [22]. However, their subjects included patients with not only asymptomatic myeloma, but also monoclonal gammopathy of undetermined significance (MGUS), which is a common premalignant plasma cell disorder, raising concern about the skewed diagnostic performance of variables in differentiating disease status. Furthermore, their assessment did not include information about correlation between fat mass derived from MRI with histopathological examination of the bone marrow.

Bone marrow biopsy is a necessary procedure for the diagnosis of various hematological diseases and is used repeatedly in patients with multiple myeloma to assess responses to therapy. However, the procedure is invasive and the associated complications have been reported at a rate of 0.05%. The most common and most serious complication is hemorrhage [23, 24] and arteriovenous fistula has also been reported [25]. In addition, evaluation by bone marrow biopsy is limited to the site of puncture in bone marrow even though several histological patterns have been found (i.e., interstitial, nodular and packed marrow patterns) in the growth of plasma cells in patients with monoclonal plasma cell disorders. Bone marrow assessment over a wide-area by fat quantification using IDEAL would provide a non-invasive radiological tool for understanding the pathogenesis of multiple myeloma and could represent a possible surrogate for bone marrow biopsy in the future.

This study aimed to evaluate the effectiveness of IDEAL MRI in discriminating between symptomatic and asymptomatic myeloma using lumbar bone marrow without visible focal lesions.

## Materials and Methods

### Ethics Statement

All study protocols were approved by the Institutional Review Board of Hiroshima University Hospital (University Hospital Medical Information Network Clinical Trials Registry [UMIN-112CTR] number, UMIN000003663). Each participant provided written informed consent before undergoing MRI.

### Study cohort

We searched a computerized database and reviewed the medical records of all patients who had been seen at Hiroshima University Hospital between July 2011 and November 2013. The criteria used for diagnosis were taken from the criteria of the International Myeloma Working Group [26]. After excluding patients with primary amyloidosis or who had undergone chemo- or radiotherapy, remaining 15 men (mean age, 60.9 years; range, 38–81 years) and 20 women (mean age, 65.5 years; range, 45–81 years) were included in the study and statistical analyses. Of these 35 patients in the staging cohort, 11 had asymptomatic myeloma and 24 had symptomatic myeloma. No patients had non-secretory myeloma. The distinction between symptomatic and asymptomatic myeloma depended on the presence or absence of myeloma-related organ dysfunction according to the criteria of the International Myeloma Working Group [26]. Myeloma-related organ dysfunction is characterized by one or more of the following clinical manifestations, denoted by the acronym CRAB: calcium elevation; renal insufficiency; anemia; and bone abnormalities, including lytic lesions and osteopenia. Of the 24 patients with symptomatic myeloma, 21 had lytic bone lesions or vertebral fractures on whole-body computed tomography (CT), 19 had anemia, and six had renal insufficiency.

Two authors (with 20 years of experience in spinal imaging and 12 years of expertise in hematology) reviewed all medical and clinical records to collect all available data.

### Spinal MRI and quantitative study

Imaging was performed using a 3.0-T MRI unit (Signa HDxt 3T; GE Healthcare Milwaukee, WI) and the following sequences (Table 1): sagittal T_1_ fast spin echo (FSE); sagittal FS-T_2_ FSE (with chemical shift selective (CHESS) technique); and a sagittal IDEAL T_2_ FSE sequence. All sequences including IDEAL are commercially available (GE Healthcare Milwaukee, WI). Co-registered water, fat, in-phase (water + fat) and out-of-phase (water-fat) images were generated using the IDEAL software. Reeder et al. [17] published detailed information about the IDEAL FSE sequence. A product version of IDEAL was used. We acquired three images at optimized phase angles of −π/6, π/2, and 7π/6 [17]. A region-growing algorithm was employed for the field-map estimation to avoid water/fat swaps as in [17]. T2* correction and the pre-calibrated multi-peak fat spectrum were not employed.

**Table 1:** Acquisition parameters for MR sequences.

Sequence	TR/TE/TI (ms)	NSA	FOV (mm)	Matrix	Slice thickness (mm)	Bandwidth (kHz)	Imaging time (min:s)
IDEAL T_2_ FSE	4000/112.4	3	300	384×192	4	83.3	5:12
Fat-suppressed T_2_ FSE	4000/116	2	300	320×288	4	62.5	2:16
T_1_ FSE	700/11.8	2	300	512×224	4	41.7	2:38

NSA, number of signal averages

Number of excitation, 2.

Mean signal intensity and standard deviation were calculated by placing operator-determined regions of interest (ROIs) within bone marrow containing no focal lesions. The ROI for BM was defined manually within the internal parts of the L1-L3 vertebral bodies because these spinal levels were less affected by degenerative disc disease compared to lower lumbar elements or less likely to be fractured compared to lower thoracic elements. ROIs for BM had an area of 254–534 mm^2^. Signal intensity values were then calculated as the mean value obtained from these three vertebral bodies (S1 Table and S1 Fig.). If one of the L1-L3 vertebrae was fractured or included a focal osteolytic lesion with a long axis larger than 0.7 cm, that vertebra was excluded from calculation of the fat-signal fraction. If two of the L1-L3 vertebrae were fractured or included a focal osteolytic lesion, that patient was excluded from this study. All focal myeloma lesions were confirmed to be shown as lytic lesions on CT. The fat-signal fraction from IDEAL images was calculated from the ratio of the signal intensity in the fat image divided by the signal intensity of the corresponding ROI in the in-phase image.

**Fig 1:**
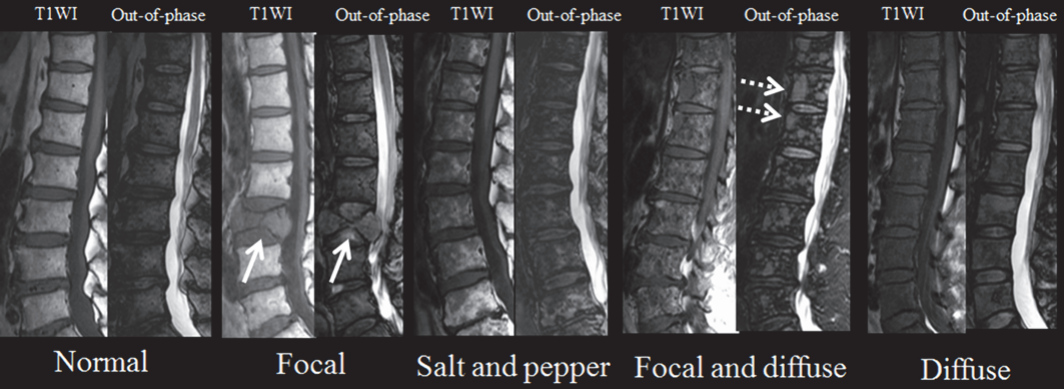
MRI of the lumbar spine shows infiltration patterns of multiple myeloma. In the focal pattern, focal areas of decreased signal intensity are seen on T1-weighted imaging. Out-of-phase image shows increased signal intensity in the corresponding areas (white arrows). In the salt-and-pepper pattern, T1-weighted imaging shows intermingled hyper- and hypointense signals. Areas of signal hyperintensity on T1-weighted imaging correspond to signal-hyperintense areas on out-of-phase images. In the focal and diffuse patterns, T1-weighted imaging shows diffusely decreased signal intensity of vertebrae and focal areas of signal hypointensity are also seen (white dotted arrows). T1WI, T1-weighted imaging.

The MR infiltration pattern was determined as follows: normal; focal; salt and pepper; focal and diffuse; or diffuse (Fig. 1). The MR infiltration pattern was defined according to the previous study by Stäbler et al [10], which showed that infiltration pattern correlated with clinical staging. The normal and diffuse pattern was defined with T1-weighted imaging, with the spinal signal compared to that from the intervertebral discs. A normal pattern was defined for a spine/disc signal ratio ≥1.3, and a diffuse pattern was defined for a spine/disc signal ratio <1.3 [27]. The second pattern comprised focal signal hypointensity on T1-weighted imaging and signal hyperintensity on out-of-phase images against a background of normal-appearing marrow. The third pattern, the salt-and-pepper pattern, was characterized by intermingled signal hyperintensity and hypointensity on T1-weighted images. Areas of signal hyperintensity on T1-weighted imaging corresponded to signal-hyperintense areas on out-of-phase images. In the fourth type, focal and diffuse infiltration patterns were combined, and signal-hypointense foci could not be readily identified within the signal-hypointense marrow on T1-weighted imaging. However, these foci were clearly visualized in out-of-phase images as bright lesions within the intermediate background marrow signal. Interrater reliability, representing the consistency of categorizations of MR infiltration patterns between raters, was measured with the k-coefficient between two raters.

### Bone marrow examination

We estimated the proportion of bone marrow plasma cells (bone marrow plasma cell percentage (BMPC%)) in bone marrow biopsy specimens obtained from the iliac crest.

### Statistical analysis

The two-tailed Student’s *t* test was used to compare continuous variables between asymptomatic and symptomatic groups. When necessary, Student’s *t*-test was modified for unequal variance with Welch’s *t*-test. These variables included: age; fat-signal fraction; BMPC%; serum monoclonal protein (M protein); albumin; β_2_-microglobulin; kappa/lambda ratio; serum creatinine; serum calcium; and hemoglobin. The χ^2^ test or Cochran-Armitage trend test were used to compare the following nominal variables: sex; MR infiltration pattern; presence of immunoglobulin (Ig) A M protein (absent vs. present); reduction of uninvolved immunoglobulin (0/1/2); and kappa/lambda ratio (0.125–8 vs. <0.125 or >8) according to the report by Dispenzieri et al. [28].

A multivariate general linear model with binomial distribution and logit-link was constructed to identify the best predictors of symptomatic myeloma. Variables showing values of *P*<0.05 on univariate analysis were included in the multivariate analysis. Mutual independence of all variables was confirmed by factor analysis with VARIMAX factor rotation.

Receiver operating characteristics (ROC) curves were constructed by plotting sensitivity (Y axis) versus 1-specificity (X axis), and the area under the curve (AUC) was calculated. The ROC curve was used to determine the ability of the variables to discriminate symptomatic myeloma from asymptomatic myeloma. Cutoff values obtained from ROC analysis were applied and the sensitivities and specificities were calculated for each parameter.

Values of P<0.05 were considered significant.

All analyses were performed using a spreadsheet application (Excel 2012; Microsoft, Redmond, WA) and ROC analysis software (ROCKIT 0.9.1; Charles E. Metz, University of Chicago, Chicago, IL).

## Results

Table 2 summarizes patient characteristics and laboratory and bone marrow findings. Patients with asymptomatic and symptomatic myeloma did not differ significantly in clinical characteristics.

**Table 2:** Characteristics of the study population.

	Asymptomatic myeloma	Symptomatic myeloma	*P* value Univariate analysis	Odds ratio
Background data				
Sex			0.8335	
Male	5	10		
Female	6	14		
Age (years)	66.5 ± 8.9	62.1 ± 11.2	0.26	
Laboratory data				
Serum creatinine (mg/dl)	0.85 ± 0.55	1.42 ± 1.52	0.06	4.44 (0.93, 21.22)
Hemoglobin (g/dl)	12.0 ± 1.26	9.76 ± 2.52	0.001	38 (3.89, 371.3)
Serum calcium (mEq/l)	4.53 ± 0.14	4.68 ± 0.59	0.41	5.33 (1.10, 25.77)
Serum M protein (mg/dl)	2858 ± 1465	3868 ± 2458	0.89	3.72 (0.79, 17.68)
M protein			0.395	
IgG	9	22		
IgA	2	2		
Uninvolved Igs			0.225	
0	1	0		
1	3	4		
2	7	20		
Albumin (g/dl)	3.70 ± 0.59	3.75 ± 1.31	0.90	2.07 (0.48, 8.97)
β_2_-microglobulin (mg/l)	2.50 ± 1.30	7.15 ± 6.62	0.003	24.3 (2.60, 227.2)
Kappa/lambda ratio			0.070	
0.125–8	5	4		
≤0.125 or ≥8	6	20		
MRI data				
MR infiltration pattern			0.04	
Normal	5	3		
Focal	0	3		
Salt and pepper	4	0		
Focal and diffuse	0	5		
Diffuse	3	12		
Fat-signal fraction	70.9 ± 8.3	47.0 ± 22.0	0.00006	9.00 (1.56, 51.87)
BM biopsy				
BMPC%	17.2 ± 9.0	37.0 ± 25.3	0.002	7.00 (1.22, 40.12)

Note: Values represent mean ± standard deviation.

Ig, immunoglobulin; Serum M protein, serum monoclonal protein level; MR pattern, MR signal intensity pattern; M protein, presence of IgA monoclonal protein; Uninvolved Igs, reduction of uninvolved Igs (number); BMPC%, bone marrow plasma cell percent.

Univariate analysis showed that β_2_-microglobulin (odds ratio (OR), 24.3; *P* = 0.003) and BMPC% (OR, 7.00; *P* = 0.02) was significantly higher, whereas fat-signal fraction (OR, 9.00; *P* = 0.002) and hemoglobin (OR, 38.00; *P* = 0.001) were significantly lower in symptomatic myeloma than in asymptomatic myeloma. A significant increase in the prevalence of symptomatic myeloma was seen from normal MR infiltration pattern to MR diffuse infiltration pattern (*P* = 0.04). Interrater variability was excellent for categorizations of MR infiltration patterns (k-coefficient: 0.85, *P* <0.001, S2 Table). No significant differences in serum M protein, albumin, kappa/lambda ratio, presence of IgA M protein, or reduction of uninvolved immunoglobulin were seen between groups.

**Fig 2:**
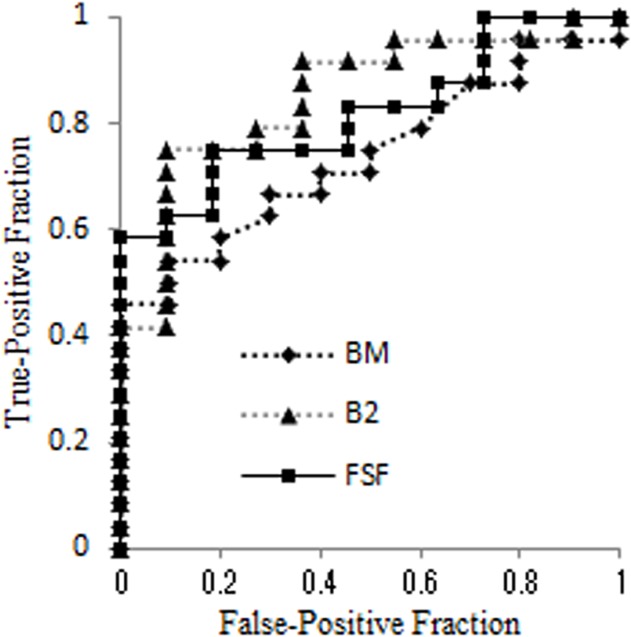
With the cutoff value derived from ROC analysis, the fat-signal fraction could be used to differentiate symptomatic myeloma from asymptomatic myeloma with 75% sensitivity and 80.2% specificity. BMPC% for symptomatic myeloma showed 66.7% sensitivity and 72.7% specificity, which resulted in an insignificant contributor to discriminate symptomatic myeloma from asymptomatic myeloma. BM, BMPC%; B2, β_2_-microglobulin; FSF, fat-signal fraction.

**Fig 3:**
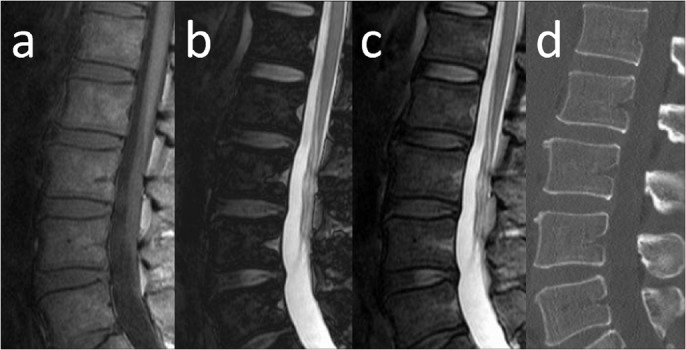
MRI of the lumbar spine in a false-positive case misclassified on the basis of β_2_-microglobulin. Sagittal MR images and sagittal reconstructed CT of a 53-year-old man with asymptomatic myeloma and a fat-signal fraction of 64% are shown. Serum creatinine level was 2.4 mg/dl, β_2_-microglobulin was 5.99 mg/l, and BMPC% from the iliac crest was 7.5%. The elevated β_2_-microglobulin level might have resulted from renal impairment. **a)** T1-weighted imaging shows diffusely decreased signal intensity in bone marrow. Bone marrow signal intensity is almost equal to that of intervertebral disc. **b)** Calculated out-phase image. **c)** Calculated in-phase image. **d)** Sagittal reconstructed CT.

**Fig 4:**
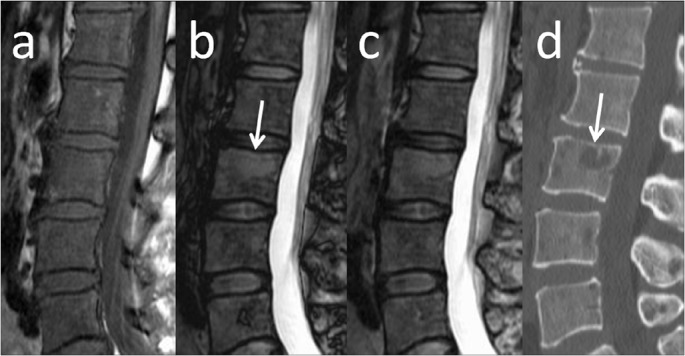
MRI of the lumbar spine in a false-negative case misclassified on the basis of β_2_-microglobulin. Sagittal MR images and sagittal reconstructed CT of a 52-year-old woman with symptomatic myeloma and a fat-signal fraction of 11% are shown. Serum creatinine level was 0.59 mg/dl, β_2_-microglobulin was 2.32 mg/l, and BMPC% from the iliac crest was 87%. The elevated β_2_-microglobulin might have resulted from renal impairment. **a)** T1-weighted imaging shows diffusely decreased signal intensity in bone marrow, suggesting replacement of fatty marrow by myeloma cells. A focal lytic lesion is also seen in the L3 vertebral body (arrows). **b)** Calculated out-phase image. **c)** Calculated in-phase image. **d)** Sagittal reconstructed CT.

Interrelationships among these variables were confirmed using factor analysis and mutual independence was confirmed. The general linear model demonstrated that fat-signal fraction and β_2_-microglobulin contributed to the risk of symptomatic myeloma (Table 3). ROC curves were used to compare the diagnostic performance to differentiate between patients with symptomatic and asymptomatic myeloma (Fig. 2, Table 4). AUCs were 0.847 for β_2_-microglobulin, 0.834 for fat-signal fraction, 0.818 for hemoglobin, and 0.759 for BMPC%. Representative images are shown in Figs. 3 and 4.

**Table 3:** General linear model examining the influence of microstructural indices for differentiating symptomatic myeloma.

Variable	*β ± standard error	Odds Ratio (95% CI)	*P*
Fat-signal fraction	-0.18 ± 0.08	0.83 (0.71–0.98)	0.024
β_2_-microglobulin	0.63 ± 0.30	1.88 (1.05–3.38)	0.035

*β, partial regression coefficient.

**Table 4:** ROC results of parameters for discriminating symptomatic myeloma.

Variables	AUC ± standard error	95% confidence interval	Sensitivity (%) †	Specificity (%) †	Cutoff
Fat-signal fraction	0.834 ± 0.069	0.67, 0.93	75.0 (18/24)	80.2 (9/11)	<63.1 (%)
β_2_-microglobulin	0.847 ± 0.065	0.68, 0.94	70.8 (17/24)	90.9 (10/11)	>3.14 (mg/l)
BMPC%	0.756 ± 0.082	0.57, 0.89	66.7 (16/24)	72.7 (8/11)	>20.0 (%)
Hemoglobin	0.818 ± 0.058	0.66, 0.90	66.7 (16/24)	100 (11/11)	<10.4 (g/dl)

† Sensitivity and specificity for identifying symptomatic myeloma. Data in parentheses represent numbers used to calculate percentages.

## Discussion

Asymptomatic myeloma is an asymptomatic precursor to multiple myeloma for which the standard of care has remained as observation without therapy until symptoms develop [3]. Early treatment of asymptomatic myeloma has been limited, due to the unacceptably high rates of treatment-related toxicity, and an inability to identify high-risk asymptomatic myeloma patients who will consistently progress to symptomatic myeloma within a short period [8, 29, 30].

In this study, fat-signal fraction and β_2_- microglobulin were identified as significant contributors to the risk of symptomatic myeloma. Furthermore, the sensitivity for symptomatic myeloma was almost identical between fat-signal fraction and β_2_-microglobulin. Beta-2 microglobulin is commonly elevated in lymphoid malignancies including multiple myeloma, showing a strong correlation to tumor burden [31, 32]. In a recent study by Rossi et al. [33] that analyzed a consecutive series of 148 patients with asymptomatic myeloma, serum β_2_-microglobulin proved to be an independent predictor of asymptomatic myeloma progression. However, serum β_2_-microglobulin not only reflects myeloma tumor load, but also increases with renal dysfunction [34]. Since 20–30% of myeloma patients present with some degree of renal dysfunction, there have been concerns that asymptomatic myeloma patients with elevated levels of β_2_-microglobulin and renal impairment do not reflect tumor burden, but rather the degree of renal dysfunction. In other words, one could expect that these patients would not meet the indications for induction of chemotherapy because of the paucity of targeted tumor volume and renal dysfunction. In fact, one patient with asymptomatic myeloma in this study was classified as false-positive on the basis of β_2_-microglobulin. That patient showed high levels of β_2_- microglobulin (5.99 mg/l) and creatinine (2. 24 mg/dl).

Serum β_2_- microglobulin level can also be considered to reflect the synergistic effects of myeloma mass and renal dysfunction. Six of the 24 patients in this study were classified as false-negative on the basis of β_2_-microglobulin. Serum β_2_- microglobulin levels were within normal limits (2.35 ± 0.52 mg/l), as were creatinine levels (0.67 ± 0.14 mg/dl). Mean creatinine level in the other 18 patients was abnormal (1.67 ± 1.23 mg/dl). These false-negative cases could result from the normal renal function that did not result in increased serum β_2_- microglobulin. Conversely, five of six false-negative patients according to β_2_- microglobulin were correctly classified with symptomatic myeloma on the basis of the fat-signal fraction. In addition, patients with non-secretory myeloma are known to be non-producers or low-producers of β_2_- microglobulin [35] and may be classified as false-negative cases.

In this study, six of 24 patients with symptomatic myeloma were classified as false-negative on the basis of the fat-signal fraction. Of these six patients, three had no bone lesions; instead they were diagnosed with symptomatic myeloma due to renal insufficiency (serum creatinine: 3.18 ± 0.75 mg/dl) and/or anemia (hemoglobin: 10.6 ± 1.9 mg/dl) using the CRAB criteria. These three patients were correctly classified with symptomatic myeloma on the basis of β_2_- microglobulin. Three other patients had focal bone lesions outside the L1-L3 vertebrae and MR signal intensities of background bone marrow on T1-weighted imaging and in-phase IDEAL images were normal on visual inspection. Two of these false-negative cases were also classified as false-negative on the basis of β_2_-microglobulin. We speculate that the false-negative results for these patients with focal MR infiltration patterns were mainly caused by reduced infiltration of myeloma cells into background bone marrow, compared to patients with focal and diffuse or diffuse patterns on MRI.

The present results demonstrate that diagnostic performance as assessed by ROC curve analysis for the fat-signal fraction is superior to that for BMPC% obtained by bone marrow biopsy. We attribute this to the wide variability of the histopathological pattern for multiple myeloma. The neoplastic plasma cells tend to form clusters, which may be small or large [36]. Single bone marrow samples thus may not necessarily represent the true status of disease. On the other hand, the fat-signal fraction of bone marrow in this study was obtained as the mean value from three vertebral bodies. This would explain the increased diagnostic performance of fat-signal fraction compared to BMPC% for identifying symptomatic myeloma.

This finding has important implications for the staging of multiple myeloma. The Durie/Salmon PLUS myeloma staging system includes stage I, which corresponds to asymptomatic myeloma. In that system, hemoglobin, serum calcium, serum M protein, serum creatinine, and number of bone lesions on MRI/positron emission tomography (PET) are used as surrogates to measure myeloma cell mass, because BMPC% by biopsy is unreliable due to the heterogeneous distribution in the skeletal system [37], as suggested in this study. The fat-signal fraction thus appears to have potential as a more appropriate marker for measuring myeloma mass in the bone marrow, although further investigation is needed of its role in stratifying patients into different risk groups for symptomatic myeloma. In addition, counting focal lesions in whole-body MRI and assessment of bone marrow infiltration using the fat-signal fraction of IDEAL can be done from a single MR examination, although whole-body MRI and fat-water quantification techniques including IDEAL are not available at all institutions.

The Durie/Salmon PLUS staging system also employs whole-body fluorodeoxyglucose (FDG)-PET as an alternative to whole-body MRI to count the number of lesions. FDG-PET/CT is an important tool for staging multiple myeloma, because of its ability to depict metabolic activity and extramedullary disease, and to evaluate the disease burden in non-secretory multiple myeloma [38]. However, if PET/CT was the sole imaging modality used, additional small skeletal lesions and diffuse spinal involvement could often be missed compared to MRI [39, 40]. However, the extent to which these small lesions detected by MRI and not by PET/CT contribute to the risk of symptomatic myeloma remains undetermined.

In previous reports [2,5], at the time of diagnosis, significant risk factors for progression have included the serum level and type of M protein, reductions in uninvolved immunoglobulins (Igs), and kappa/lambda ratio. These items did not show any significant difference between patients with symptomatic and asymptomatic myeloma in the present study. We believe this finding may have resulted from our cross-sectional study design and the relatively small number of patients, although similar trends were seen between these two statuses in our cases.

In this study, 5 of 24 symptomatic myeloma patients were categorized as showing the focal and diffuse MR infiltration pattern. In this pattern, focal lesions with low signal intensity cannot be readily identified on T1-weighted imaging because of the background signal-hypointense marrow. However, these foci were clearly visualized in out-of-phase images as bright lesions within the intermediate background signal from marrow. In the report by Stäbler et al [10], focal accumulations of myeloma cells displayed high signal intensity on out-of-phase images. On the other hand, bone marrow with nearly equal amounts of fat- and water-containing cells such as normal hematopoietic marrow was seen as areas of low signal intensity on sequences with subtraction of the transverse magnetization of fat and water, i.e., out-of-phase images [41]. We therefore, speculated that moderate myeloma cell infiltration into bone marrow interstitium, resulting in mix of fat cells and water-containing tumor cells, caused a diffuse decrease in the signal intensity of bone marrow on out-of-phase images, making focal lesions conspicuous.

This study has several limitations. The most notable was the small number of patients included, which obviously restricted the statistical analyses we performed and thus could have impaired the explanatory power for discriminating the status of the disease. We attempted to include as many patients as possible, but the need to achieve an adequately homogenous sample of patients required the exclusion of some patients. The limited number of patients included also led to another limitation, prohibiting subgroup analyses and thus preventing insights into risk stratification for progression from asymptomatic myeloma to symptomatic myeloma. Second, we did not implement advanced corrections for relaxation and noise bias in the IDEAL method [42, 18, 43]. We did not use a small flip angle for the excitation RF pulses to approach proton-density weighting, which may have caused quantification errors. Likewise, corrections of other confounding factors such as T2* relaxation, multiple resonance peaks of fat, and noise bias would ideally be integrated into the IDEAL for more accurate fat-signal fraction measurement [43, 44]. Third, no histopathological evaluation was performed for spinal bone marrow in this study. Factors including hematopoietic bone marrow and degenerative disc disease might thus have decreased the fat-signal fraction of bone marrow. In contrast to yellow marrow, which contains predominantly fat, hematopoietic bone marrow is characterized by similar proportions of water from hematopoietic cells, extracellular fluid, connective tissue, and fat. Hematopoietic bone marrow consequently exhibits marked microscopic field inhomogeneities due to the bony trabecular structure and increased iron deposition. With advancing age, the hematopoietic BM becomes increasingly replaced with fatty marrow [45]. The BM also undergoes changes due to dietary changes, anemia, chronic hypoxia, chemotherapy, and other medications [46, 47]. Such interindividual variability of fatty marrow replacement could thus have affected our results. Although we selected the ROI within the internal parts of the vertebral bodies to avoid degenerated endplates, some error in the quantification of signal intensity might have occurred. BMPC% obtained from lumbar bone marrow would show better discriminating power between asymptomatic and symptomatic myeloma, although biopsy of a vertebral body for all patients is difficult to perform in the clinical setting. Fourth, fat quantification with MRI in our study was limited to lumbar vertebrae. As described before, because skeletal involvement in multiple myeloma shows highly variable distribution, the use of specific vertebral bodies might not be appropriate for assessing all bone marrow in the body. Third, we defined MR infiltration patterns using T1-weighted and out-phase images with the IDEAL sequence, instead of using the opposed gradient-recalled echo images described by Stäbler et al. [10]. By two-echo methods to separate water- and fat MR signals with symmetrical sampling to yield in- and opposed-phase data, identification of water and fat can be ambiguous (water-fat swaps) due to B_0_ inhomogeneity [48]. In three-echo methods including IDEAL, asymmetrical sampling can overcome this ambiguity to achieve consistent water-fat separation and identification [49, 50]. Categorization of MR infiltration patterns might thus be somewhat different from that of their study due to, for example, differences in the delineation of yellow marrow.

In conclusion, the-fat signal fraction as a biomarker for multiple myeloma enables discrimination of symptomatic myeloma from asymptomatic myeloma. The sensitivity and specificity of the fat-signal fraction were superior to those of BMPC% from biopsy specimens. This finding may be related to the fact that skeletal involvement by multiple myeloma shows heterogeneous distribution. The diagnostic performance of the fat-signal fraction and β_2_-microglobulin were comparable for assessing the risk of symptomatic myeloma. Some false-negative cases assigned by the fat-signal fraction were diagnosed as symptomatic myeloma by items other than skeletal lesions in the CRAB criteria. A larger, longitudinal study would allow estimation of the progression of asymptomatic myeloma into symptomatic myeloma using the IDEAL sequence in patients with multiple myeloma.

## Supporting Information

S1 Fig MRI of the lumbar spine for calculation of the fat-signal fraction is shown. The volumes of interest are defined manually within the internal part of the L1 to L3 vertebral bodies. L1 to L3, L1 to L3 vertebral body, respectively; T1WI, T1-weighted imaging; Water image, water image of IDEAL
(TIF)

S1 Table Signal intensity values of three vertebral bodies are shown. FSF, fat-signal fraction; L1 to L3, L1 to L3 vertebral body, respectively
(XLS)

S2 Table Categorizations of MR infiltration patterns between raters. (DOC)
