# Supplementary figures and images for: Iterative Decomposition of Water and Fat with Echo Asymmetry and Least-Squares Estimation (IDEAL) Magnetic Resonance Imaging as a Biomarker for Symptomatic Multiple Myeloma
Source: PLoS One. 2015 Feb 23;10(2):e0116842. doi: 10.1371/journal.pone.0116842 (PMC4338220; doi:10.1371/journal.pone.0116842)

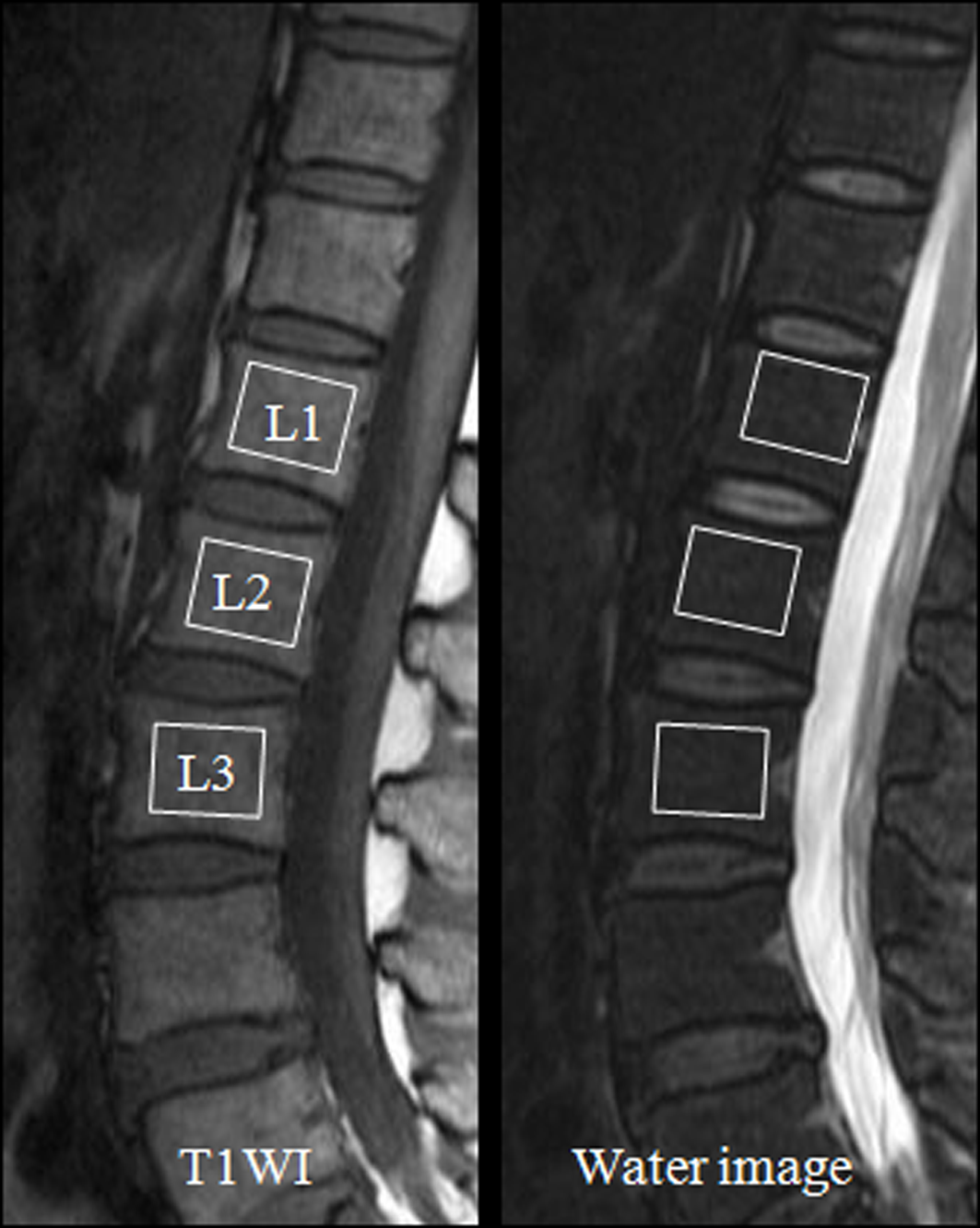

Supplement: S1 Fig — The volumes of interest are defined manually within the internal part of the L1 to L3 vertebral bodies. L1 to L3, L1 to L3 vertebral body, respectively; T1WI, T1-weighted imaging; Water image, water image of IDEAL (TIF) [file pone.0116842.s001.tif]
