# Supplementary material for: Iterative Decomposition of Water and Fat with Echo Asymmetry and Least-Squares Estimation (IDEAL) Magnetic Resonance Imaging as a Biomarker for Symptomatic Multiple Myeloma
Source: PLoS One. 2015 Feb 23;10(2):e0116842. doi: 10.1371/journal.pone.0116842 (PMC4338220; doi:10.1371/journal.pone.0116842)
Supplement: S2 Table — (DOC) [file pone.0116842.s003.doc]

**S3 Table.** Categorizations of MR infiltration patterns between raters.

|  | Normal | Focal | SP | FD | Diffuse |
| --- | --- | --- | --- | --- | --- |
| Normal | 5 | 0 | 2 | 0 | 0 |
| Focal | 0 | 4 | 0 | 0 | 0 |
| SP | 0 | 0 | 4 | 0 | 0 |
| FD | 0 | 0 | 0 | 5 | 0 |
| Diffuse | 0 | 0 | 2 | 0 | 13 |

SP, salt and pepper; FD, focal and diffuse.
